# Role of Protein Glycosylation in Host-Pathogen Interaction

**DOI:** 10.3390/cells9041022

**Published:** 2020-04-20

**Authors:** Borong Lin, Xue Qing, Jinling Liao, Kan Zhuo

**Affiliations:** 1 Laboratory of Plant Nematology, South China Agricultural University, Guangzhou 510642, China; boronglin@scau.edu.cn (B.L.); jlliao@scau.edu.cn (J.L.); 2 Guangdong Province Key Laboratory of Microbial Signals and Disease Control, South China Agricultural University, Guangzhou 510642, China; 3 College of Plant Protection, Nanjing Agricultural University, Nanjing 210095, China; qingxue@njau.edu.cn; 4 Guangdong Eco-Engineering Polytechnic, Guangzhou 510520, China

**Keywords:** glycoprotein, host, interaction, pathogen, resistance, virulence

## Abstract

Host-pathogen interactions are fundamental to our understanding of infectious diseases. Protein glycosylation is one kind of common post-translational modification, forming glycoproteins and modulating numerous important biological processes. It also occurs in host-pathogen interaction, affecting host resistance or pathogen virulence often because glycans regulate protein conformation, activity, and stability, etc. This review summarizes various roles of different glycoproteins during the interaction, which include: host glycoproteins prevent pathogens as barriers; pathogen glycoproteins promote pathogens to attack host proteins as weapons; pathogens glycosylate proteins of the host to enhance virulence; and hosts sense pathogen glycoproteins to induce resistance. In addition, this review also intends to summarize the roles of lectin (a class of protein entangled with glycoprotein) in host-pathogen interactions, including bacterial adhesins, viral lectins or host lectins. Although these studies show the importance of protein glycosylation in host-pathogen interaction, much remains to be discovered about the interaction mechanism.

## 1. Introduction

Glycosylation is the process by which a carbohydrate is attached to a target macromolecule, typically proteins and lipids. In respect to proteins, this process is called “protein glycosylation” and the newly formed molecule is called a “glycoprotein” [1]. Protein glycosylation is a widespread post-translational modification that is conserved among various organisms and most cells of a specific individual [2]. Such a process plays a significant role in protein folding, targeted transport, cellular localisation and activity [3]. In an organism, glycoproteins are involved in multiple biological processes: cell recognition, differentiation, development, signal transduction, and immunity response, etc. For pathogen-host interactions, the pathogenicity, on the one hand, and host resistance (susceptibility) on the other, can be attributed to the glycosylation, for instance, pathogen-secreted proteins, host cell-surface, and organelle-resident proteins [4]. Glycoproteins are found in various forms, e.g., enzymes (nuclease, protease, glycosidase) [5,6], peptide hormones (chorionic gonadotropin, luteinizing hormone, thyrotropin, erythropoietin) [7], antibodies [8], lectins [9], membrane bond proteins [10], collagen [11], and fibronectin [12], some of which have great significance for clinical medicine. Those glycoproteins discussed in this review, with roles in host-pathogen interactions, are listed in Table 1.

Due to the structural variability of carbohydrate side chains, glycosylation increases the diversity of the proteome to a level unmatched by any other post-translational modifications [2]. While carbohydrate is universal in nature, only a few monosaccharides can serve as a donor in glycosylation. The main substrates in the protein glycosylation are hexose and its derivatives, including d-glucose [43], d-mannose [44], d-galactose [45], fucose [46], *N*-acetyl-glucosamine [47], and galactosamine [48]. In some proteins, sialic acid (SA) or xylose are also the substrates of protein glycosylation [49,50]. During glycosylation, straight or branched oligosaccharides consisting of 2–20 monosaccharide units are covalently bonded with amino acid side chains in proteins to form glycoproteins, catalyzed by glycosyltransferases which located in the endoplasmic reticulum (ER) and Golgi complex [51].

**Figure 1:**
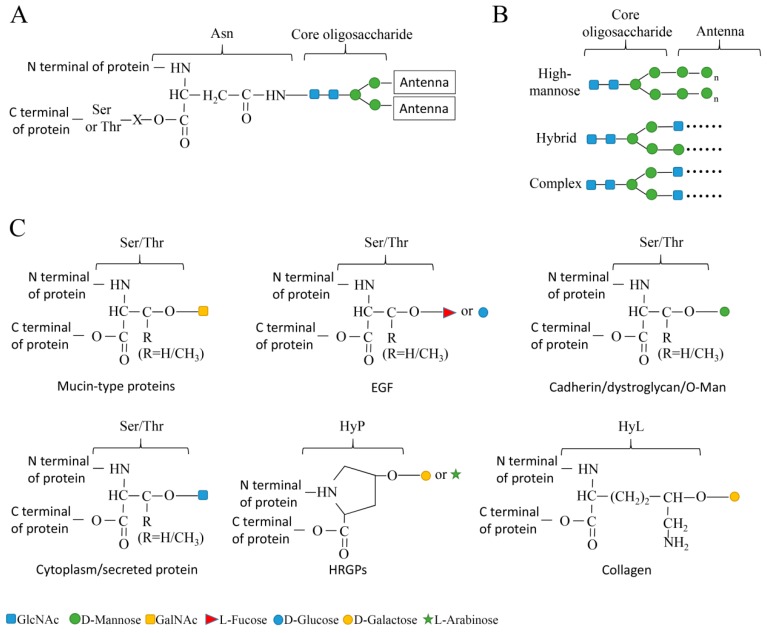
Diagram showing the structure of *N*-linked and *O*-linked glycosylation types. (**A**) *N*-linked glycoprotein. The core oligosaccharide links to the amide nitrogen of asparagine (Asn) in the consensus sequence Asn-X-Ser/Thr (Ser, Thr, and X represent the serine, threonine and random amino acids). (**B**) Different subtypes of *N*-glycoproteins. High-mannose, oligosaccharide chains linked to the core oligosaccharide via mannose; Hybrid, oligosaccharide chains linked to the core oligosaccharide via GlcNAc and mannose; Complex, oligosaccharide chains linked to the core oligosaccharide via GlcNAc. (**C**) *O*-linked glycoprotein, oligosaccharides are bonded to the hydroxyl of Ser, Thr, hydroxylysine (HyL) or hydroxyproline (HyP) of proteins. Although the *O*-linked glycoprotein has no core oligosaccharide, constant protein-glycans linkage was found in some types of proteins, such as GalNAc-Ser/Thr linkage found in Mucin-type proteins, l-fucose- or d-glucose- Ser/Thr linkage found in epidermal growth-factor-like repeats (EGF), d-mannose- Ser/Thr linkage found in Cadherin, dystroglycan or fungi and prokaryotes glycoproteins (Cadherin/dystroglycan/O-Man), GlcNAc- Ser/Thr linkage found in nucleocytosol glycoproteins or secreted glycoproteins in eukaryotic cells except yeast (Cytoplasm/secreted protein), d-galactose- or l-arabinose- HyP linkage found in hydroxyproline-rich glycoproteins (HRGPs) and d-galactose- HyL linkage found in Collagen.

According to the amino acid atom to which the carbohydrate chain is linked, the glycoproteins can be divided into *N*-linked and *O*-linked glycoprotein subtypes [2] (Figure 1). Protein modification by structurally varying glycosylation can either be favourable or disadvantageous for the infected organism, as it affects both, the host resistance and the pathogen virulence. In this review, we summarize the roles of protein glycosylation in pathogen-host interactions.

## 2. Host-Pathogen Interactions

Host-pathogen interactions (HPIs) are complex and dynamic in nature and crucial for our understanding of infectious diseases. Most studies on HPIs primarily focus on how pathogens attack and exploit hosts, e.g., how pathogen invades hosts, evades from host defense, and proliferates in hosts, and in turn how host protects themselves against pathogens, e.g., how host immune responds to invaders [52].

Pathogen invasion starts from penetration into the host. Several ways of entry or pathways can be used, such as entering or (and) adhering to wounds on the host (cells) surface, mucosa membrane or natural orifices [53,54]. After entering hosts, pathogens interact with host cells for their survival and proliferation, with different targets and in specific mechanisms. For instance, viral pathogens invade host cells by merging their envelope with the membrane of host cells [55]; parasitic nematodes, fungi or bacteria can use special secretion systems to release effectors into host cells, manipulating some physiological and biochemical processes in hosts [56,57,58]; fungi also can utilize effectors on their hyphae surface to target host cell receptors and induce the endocytosis or cellular uptake of pathogens [59]. Pathogens may also interact with the host immune system, including the triggering of host immune responses and the pathogen evasion from these. Host immune responses are stepwise, first triggered by pathogen conserved molecules recognized by host-specific receptors. Then, through signal pathways, the defense signals are transmitted to the out- or in-side of cells. Finally, hosts receive the defense signals and then activate defense responses to tackle pathogens. Pathogens might attack any defense steps, they can evade recognition by host receptors through changing or masking their conserved molecules [60,61,62,63]. Invaders also may sabotage defense responses through disturbing the activity of key proteins or degrading signal molecules, which are related to a variety of defense responses or function in inhibiting the growth of pathogens or killing pathogens directly [59,64,65].

Proliferation is essential for a successful pathogen invasion. This process is normally accomplished by manipulating the host cellular machinery in different ways. The acellular pathogens, like a virus, viroid or prion, usually utilize nucleic acid or host protein synthesis pathways to replicate itself [66,67]; while cellular pathogens, such as fungi, bacteria, protozoon or nematodes, gain only nutrients from hosts but utilize their own cellular machinery for proliferation [58,68]. In this scenario, they need to secrete effectors into hosts, altering the host energy metabolism pathway or transferring nutrients to the infection site.

On the whole, most of these processes can be mediated via protein-protein interactions, including cell surface protein interactions between hosts and pathogens, host cell surface protein and pathogen effector protein interactions, host-secreted protein and pathogen effector protein interactions, and host-secreted protein and pathogen cell surface protein interactions. These proteins often undergo glycosylation during their maturation, playing a significant role in host-pathogen interactions. For example, epithelial human blood group antigens (HBGAs) on *O*-glycans function in adherence and invasion of pathogens [69]. Mucin MUC5AC, which is glycosylated by HBGAs (Lewis b), can promote *Helicobacter pylori* infection, but mucin MUC6 glycosylated by GlcNAc-capped glycans, can inhibit *H. pylori* infection [16,33]. In general, protein glycosylation in host strongly impacts pathogen binding and invasion [70,71,72].

## 3. Glycosylated Proteins of Hosts Act as Barriers to Defense Pathogens

Animals and plants are exposed to an array of pathogens, but only a few can actually cause severe diseases [73,74]. This is because the attack of most pathogens is blocked by glycosylated proteins covering the host cell surface as barriers. Such barriers may act in two manners in host defense, (I) physically separate pathogens and host cells [75,76]; (II) chemically inhibit or kill pathogens [16,77,78,79].

### 3.1. Physical Barrier

In animals, the physical contact of the pathogen to epithelial cells can be prevented by a rigid or visco-elastic gel formed by secretory oligomerized mucins [80]. Heavily *O*-glycosylated mucins forming a dense mucus layer can prevent the attachment of parasitic nematodes (e.g., *Nippostrongylus brasiliensis*) [81]. In contrast, after the mucus layer was removed by mustard oil, cysteine protease or papain, the intestinal colonization of *N. brasiliensis* was increased [80,82]. Likewise, the study of *Trichuris muris* infection in mice suggests that expression of the mucins was exclusively induced in *T. muris* resistant mice. These mucins promoted *T. muris* to be expelled from intestinal tracts, and accordingly, a knock-out of the mucin gene abolished protective functions in the host [15]. A similar protective phenomenon was also observed during the infection of *Trichostrongylus colubriformis*, *Trichinella spiralis, Helicobacter pylori* and *Hymenolepis diminuta* [16,83,84,85]. In addition, cell surface mucins like MUC1 contribute to the formation of the apical cell glycocalyx in wet-surfaced epithelia, inhibiting the infection of *Corynebacteria* and coagulase-negative *Staphylococci* [86]. It’s worthy to note that mucins, especially secretory mucins also involve in promoting pathogen infection via adhesions on the pathogen, as discussed in Section 7 (lectin, a class of protein entangled with glycoprotein, affects host-pathogen interactions).

In plants, hydroxyproline-rich glycoproteins (HRGPs) which are characterized by a rigid polyproline type II (PPII) conformation and extensive *O*-glycosylation of 4 (R)-hydroxy-l-proline residues [87], can physically inhibit pathogen penetration [88]. HRGPs play a leading role in plant defense, e.g., HRGPs were induced in incompatible plant-pathogen interactions [18,89] and rapidly deposited in the cell wall after pathogen infection [90,91]. Such a defense ability is a result of the intra- and inter-molecular cross-linking in plant cell walls after HRGPs become insolubilized [88,92]. In this process, the interaction between insoluble HRGPs and the polysaccharide reduces the hydration and pectin mobility of the cell wall thus leading to the increase of rigidity and resistance of cell wall against pathogen invasion [92]. Furthermore, it was found that intra- and inter-molecular cross-linking took place in glycosylated HRGPs under the catalysis of the peroxidase. However, the deglycosylated HRGPs could not be cross-linked [18], suggesting that glycosylation contributes to the HRGPs network formation.

### 3.2. Chemical Barrier

In animals, the glycoproteins facilitate the growth of probiotics in the gut thus provides a chemical barrier against pathogens [93]. The *O*-glycans of glycoproteins like MUC2 can serve as attachment sites for probiotic colonization [13,94] by providing favorable nutrition substances [95]. Consequently, intestinal surface harbors a complex ecosystem consisting of a myriad of intestinal microorganisms that affect the physiology, immune function, and fitness of the host [96]. Among those probiotics including *Bifidobacterium* and *Lactobacillus* are considered as the key players to exert health-promoting effects [94].

Glycoproteins can also eliminate gut pathogens comparable to antibiotics [16]. *Helicobacter pylori* is a severe pathogen that infects the stomachs of nearly half of human population. It widely presents in stomachs but rarely in deeper portions of the gastric mucosa that coated by the mucus [97,98]. Kawakubo et al. [16] discovered that the special *O*-linked carbohydrate terminal, α(1,4)-GlcNAc, and the mucins (comprising of Muc6) with α(1,4)-GlcNAc expressed in the gastric mucosa have activity against *H. pylori* by inhibiting the biosynthesis of the cell wall.

In plants, glycosylation can stabilize plant extracellular proteinases and regulate their activities against pathogen invasion. Usually, the active sites of plant extracellular proteinases are protected by *N*-carbohydrate residues during glycosylation to avoid the proteolytic process introduced by other proteases [5]. For example, the aspartic proteinases (StAPs) are extracellular proteinase from potato which have in vitro antifungal activity towards *Phytophthora infestans* and *Fusarium solani* [19,77,99]. In apoplast StAPs accumulated significant more in the *P. infestans* resistant cultivar than in susceptible cultivar [19], accumulated slower when unglycosylated than glycosylated, and the fungicidal activity of deglycosylated StAPs was lower than native StAPs [5].

## 4. Glycosylated Proteins of Pathogens Act as Weapons to Attack the Host

Glycosylated proteins are involved in both pathogen infection and host defense to pathogens. In pathogens, cell surface proteins and secretory proteins are the main glycoproteins that can promote infection.

### 4.1. Cell Surface Glycoproteins

Host cell adhesion is important for infection initiation, and such a process is mediated by pathogen cell surface glycoproteins [100,101,102,103]. One example is the cell surface glycoprotein HMW1 in *Haemophilus influenzae*. It can mediate bacterial adhesion by interacting with a specific receptor on the human cell surface [104]. The activity of HMW1 relies on the other two proteins HMW1B and HMW1C, the former is inserted into the bacterial outer membrane, mediating the HMW1 to display on the cell surface [105], while the latter with glycosyltransferase activity can catalyse glycosylation of HMW1 in the cytoplasm [20]. The HMW1 is tethered to the bacterial surface by the glycans. However, deglycosylation HMW1 sheds from the bacterial cell membrane. The deficiency of HMW1 inhibits bacterial adhesion to host epithelial cells, suggesting HMW1 is bound to the bacterial surface only after glycosylation [101].

### 4.2. Secreted Glycoproteins

Increasing secreted proteins, or called effector, contribute to the virulence of pathogens [25,106]. Some effectors must undergo glycosylation to activate their function. A typical example is LysM Protein1 (Slp1) secreted by the rice blast fungus *Magnaporthe oryzae*. Slp1 is a critical virulence factor for *M. oryzae* that can bind with the fungal pathogen-associated molecular pattern (PAMP) chitin to escape the PAMP perception by the rice (*Oryza sativa*) chitin elicitor binding protein (CEBiP), such that rice blast fungus can successfully evade host innate immune [21]. The activity and stability of the chitin-binding of Slp1 depend on the existence of *N*-glycosylation. Specifically, Slp1 is unable to bind chitin and degrades significantly faster without glycosylation. Nonglycosylated Slp1 results in higher free chitin build-up that is more recognizable by CEBiP and, in turn, triggering plant innate immunity responses [22]. Other effectors like BAS4 (from *M. oryzae*) [22], CBH1 (from *Trichoderma reesei*) [23], and PCIPGII (from *Phytophthora capsici*) [24] also confirm *N*-glycosylation as an essential factor for pathogen virulence.

The glycans are covalently linked to effectors within the fungus cells. However, effectors from plant-parasitic nematodes can recruit host post-translational machinery rather than their own to fulfill glycosylation. As an example, the effector MgGPP secreted by the rice root-knot nematode *Meloidogyne graminicola* promotes parasitism by suppressing plant defenses, but only validate after *N*-glycosylation. Interestingly, this *N*-glycosylation occurs in the ER of host cells instead of *M. graminicola* cells [25]. Similarly, the effector GrCLE in the cyst nematode *Globodera rostochiensis* is also glycosylated using the host cellular machinery, following secretion into plant cells. The arabinosylation of the GrCLE is crucial for successful parasitism by binding a CLAVATA2-like receptor (StCLV2) from potatoes to regulate plant development [26].

Regardless the limited evidence available, plant-parasitic nematodes are unlikely to *N*-glycosylate effectors by their own post-translational machinery for three possible reasons: (I) no glycosyltransferase is expressed in esophageal gland cells (the key effector’s generator in plant-parasitic nematodes); (II) there is no appropriate glycan donor in esophageal gland cells; (III) the consensus domains in *N*-glycosylation proteins from plant-parasitic nematodes are unique. Therefore, the effector is likely to be *N*-glycosylated in host cells rather than ER-Golgi apparatus in esophageal glands. This special mechanism in plant-parasitic nematodes may have evolutionary merit in evading host immunity.

## 5. Proteins of the Host Are Glycosylated by Pathogens to Enhance Virulence

Pathogens are capable of glycosylating self-proteins and host proteins to enhance their virulence. Toxin A (TcdA) and toxin B (TcdB) are two predominant virulence factors of the bacillus *Clostridium difficile,* they can glycosylate the host Rho GTPases which otherwise cannot be glycosylated [107]. Both TcdA and TcdB contain a glucosyltransferase domain in N-terminal, conferring cytopathic and cytotoxic effects in intoxicated host cells [108,109]. TcdA/B enters into host cells through endocytosis [27], selectively modifies the host Rho GTPases by mono-*O*-glucosylation, and subsequently cause inhibitory effects [109,110]. This interaction is remarkable since Rho GTPase is the key member involved in various biological processes and signaling pathways [111]. The inactivation of Rho GTPase would block signal transmissions and regulatory functions, and resulting in cell morphological changes [112], apoptosis [113], phagocytosis dysregulation [114], pseudomembranous colitis and antibiotic-associated diarrhea in hosts [107].

## 6. Hosts Sense Glycoproteins of Pathogens to Induce Resistance

Pathogen glycoprotein is used both by pathogens and hosts. It can facilitate infection by increasing pathogen pathogenicity but also triggering the host immune response. During the invasion of parasitic helminthes, proteases are secreted [115] and glycosylated to activate their function in degrading multiple host defensive proteins, and subsequently suppress and modulate host immune response [28]. Cysteine protease (Hc-CPL-1) is essential for the parasitism of *Haemonchus contortus*. Interestingly, Hc-CPL-1 is recognized by the host protective antibody to induce immunity [116]. Recombinant cysteine protease (rHc-CPL-1) that expressed in *E. coli* only induces weaker immunity responses in sheep than the original Hc-CPL-1 [117]. Previous studies have reported that carbohydrate chains are covalently linked in original HcCPL-1 but absent in rHc-CPL-1. The host protective antibody mainly recognized glycan epitopes for triggering defenses [117], showing that the glycosylation of Hc-CPL-1 plays a major role in parasite recognition. Similarly, glycoproteins of other pathogens, like hemagglutinin of influenza viruses [31], glycoprotein E1 of alphavirus [30,118] and envelope protein (gp120) of HIV [29], are also recognized by hosts to inhibit pathogen invasion. They all can be bound by the host retrocyclin 2 (RC2) to inhibit virus infection, but the deglycosylation form can’t be bound by RC2 [31].

## 7. Lectin, a Class of Protein Entangled with Glycoprotein, Affects Host-Pathogen Interactions

The first lectin is ricin identified in protein extracts from castor seed in 1888 [119]. After that, more than 1000 similar proteins have been found in animals, plants, and microorganisms. Most of these proteins can agglutinate erythrocyte and named as lectin [120]. Lectin usually contains at least one non-catalytic carbohydrate-binding domain (CBD). It can reversibly and specifically bind free sugars or glycans of glycoprotein and glycolipid (a lipid that contains carbohydrate radical) while keeping the structure of the glycans [102,119]. Lectins are placed in a super heterogeneous protein family, various in molecular structure, carbohydrate-binding specificity, and biological activities. The validation of the current classification system of lectins is subject to discussion. The two widely used lectin classification systems are either based on the sugar-binding property or on the structure of CBD. In the first system, lectins are grouped as mannose-binding lectins, galactose-binding lectins, and chitin-binding lectins. However, this system is problematic, as lectins prefer oligosaccharides or complex glycans than monosaccharides, and it ignores evolutionary or sequence relationships. The CBD based system emerges and gradually becomes the key for lectin classification. The CBD is characterized by its specific amino acid sequence, typical folding of the lectin polypeptide, and the binding site structure. In this system, lectins can be split into at least 24 families. Among them 12 present in animals and 12 present in plants (Table 2) [99,100]. Besides, lectins can also be assigned based on the domain architecture, resulting merolectins (contain a single CBD), hololectins (contain two or more same CBDs), chimerolentins (contain CBD(s) and other domains independent from lectin domain) and superlectins (contain two different CBDs) [99].

Lectins have a variety of functions in vivo, such as participating in host-pathogen interaction [72,182], inhibiting nutrition absorption [189], intercellular recognition [190], cell migration [191] and signal transduction [111]. These functions rely on the sugar-binding activity of lectins. During the host-pathogen interaction, lectins have an entangled relationship to glycoproteins. Except for some cases (e.g., capsid proteins of noroviruses [59], chitinase-like lectins [160,161] and galectins [192]), many immature lectins are covalently bound to the glycans. Although their exact role remains unknown, research has suggested glycans can promote the CBD stability [22]. After that, mature lectins can further bind other sugar or glycans via van der Waals forces [27]. The main role of glycans on proteins emerges through their interactions with lectins, like those on bacteria (e.g., *Helicobacter pylori* or *Salmonella enteritidis*) or on virus (e.g., influenza virus or human immunodeficiency virus).

### 7.1. Pathogen Lectins

Pathogen lectins usually promote infection by sensing hosts or suppressing host immunity. Host recognition is the prerequisite of a successful pathogen infection [193]. This process is affected by a variety of factors, among which lectins on the surface of pathogens and glycans on the surface of host cells (*N*-linked or *O*-linked carbohydrate residues of glycolipids or glycoproteins) are crucial [194]. One typical example is the Influenza Virus (IAVs) surface glycoprotein haemagglutinin (HA). It is a trimeric lectin in IAVs that can help host recognition and further cytoplasm penetration through binding SA-containing glycoproteins on host cell membranes [195,196]. The subtypes of IAVs vary in host ranges depend on specific host recognition of HA [197], especially the differences in the glycosidic bonds of penultimate carbohydrate of the SA residue [198,199]. In avian respiratory epidermal cells, the linkage of penultimate carbohydrate of the SA residues is α-2,3 glycosidic bonds, while it is α-2,6 glycosidic bonds in human [200]. Based on amino acid sequences in the HA sugar-binding domain, avian-adapted IAVs preferentially bind α-2,3-SA while it is α-2,6-SA for human-adapted IAVs [201,202]. Another example is the gram-negative bacterium *Pseudomonas aeruginosa*, which can cause various diseases in humans, such as bacteremia, chronic pulmonary infection, urinary tract infection and acute ulcerative keratitis. It has two lectins, LecA and LecB, respectively binding the galactose and fucose residues of glycoconjugates on epithelial cells of various tissues. With these two lectins, *P. aeruginosa* can recognize and infect the lung or other host cell tissues [32]. Another gram-negative bacterium, *H. pylori* that causes gastritis, stomach ulcers, and stomach cancer, has different lectins on the cell surfaces [203,204]. These lectins recognize the carbohydrate residues on the cell surface of the gastric mucosa to mediate *H. pylori* infection. Among several lectins in *H. pylori* outer membrane, the blood group antigen-binding adhesin (BabA) and the lacdiNAc-binding adhesin (LabA) are well studied. BabA binds fucosylated residues of Lewis b antigen and H1 antigen, and LabA binds N,N’-diacetyllactosediamine residues, these two residues are carried by the mucin MUC5AC in human gastric surface epithelia [17,33]. Magalhaes et al. demonstrated that α(1,2)fucosyltransferase deficient mice resulted in the inhibition of the fucosylation of Lewis b, leading to the significant reduction of *H. pylori* infection [205].

The pathogen lectins can also suppress the immunity of hosts. A notable example is the C-type lectin. C-type lectin is a class of animal-specific lectin that can possess the Ca^2+^-dependent sugar recognition activity on the cell surface. Several C-type lectins can be secreted by animal-parasitic nematodes (APNs): Tc-CTL-1 and Tc-CTL-4 secreted by *Toxocara canis*, CTL1/2 released by *Heligmosomoides polygyrus* and *Nippostrongylus brasiliensis* [206,207,208]. These C-type lectins share the similar sequence to the immune cell lectins in their mammalian hosts, suggesting these proteins are involved in distinct roles in immune evasion [209]. Moreover, the pathogen-secreted lectins can mask the PAMP chitin from the cell wall by binding the oligosaccharides to evade immune surveillance of hosts, and these lectins include Slp1 from *Magnaporthe oryzae* [21], Ecp6 from *Cladosporium fulvum* [34], and LysM1 from *Trichophyton rubrum* [35]. Alternatively, pathogen-secreted lectins might also suppress the host immune response by disturbing the sucrose signal. The lectin calreticulin [210,211] Mi-CRT secreted by *Meloidogyne incognita* [212] can inhibit the PAMP-triggered immunity (PTI) elicited by ELF18 that derives from the N-terminal of bacterial elongation factor-Tu. The C-type lectin Mg01965 secreted by *M. graminicola* can suppress the PTI induced by flg22 which is a PAMP from the N-terminal of bacterial flagellin [131]. The above two lectins are unlikely to mask oligosaccharides, since carbohydrate residues are absent in ELF18 and flg22. In plants, endogenous sugar or sugar complex often involves in signal transduction. Indeed, local increases in certain sugar levels have been found under biotic stresses [213,214,215]. Shifts in apoplastic/cell sugar content may be sensed by plants and lead to the induction of immunity [215]. Therefore, the pathogen lectins may affect the signal transduction through binding endogenous sugar or sugar complex to suppress the immune responses [216,217].

### 7.2. Host Lectins

Host lectins are normally beneficial in pathogen resistance through inhibiting pathogen growth and reproduction or inducing the host immune responses. The animal or plant lectins can inhibit the development of various pathogens including fungi, bacteria, and parasites. For example, a lectin with hemagglutinating activity extracted from *Phaseolus vulgaris* can inhibit the growth of the fungi *Valsa mali* [218]; the lectin PSL isolated from *Pisum sativum* seeds exhibits antifungal activity against *Aspergillus flavus, Trichoderma viride* and *Fusarium oxysporum* [36]; a chitin-binding lectin isolated from *Solanum tuberosum* shows antibacterial activity against *Listeria monocytogenes*, *E. coli*, *Salmonella enteritidis* [219]; the lectin BpLec from *Bothrops pauloensis* snake venom decreases replication of the parasite *Toxoplasma gondii* [37]. Host lectins can bind peptidoglycans, polysaccharides, lipopolysaccharides (LPSs) or teichuronic acids on the pathogen surface to disturb the cell wall function and thus restrain pathogens [220].

Membrane-bound lectins of hosts can recognize pathogens by the glycans on the pathogen surface, and transmit signals to trigger immune responses. One example is the mincle that can mediate immune responses to resist infection from various pathogens [221]. In mice, the mincle participates in the immune response against *Candida albicans.* The cell wall of *C. albicans* contains α-mannosyl or *N*-acetylglucosamine residues with specific geometry. It can be specifically bound by a common carbohydrate-binding domain consisting of Glu-Pro-Asn that presents in mice. These glycan residues activate mouse mincle and trigger the Syk/Card pathway to effectively activate Nuclear Factor kappa-B (NF-κB) signaling. Such a process increases the cytokine expression levels, and consequently prevents the *C. albicans* infection [38,222].

In some cases, however, host lectins can also be deleterious when it is used by pathogens. Different from the aforementioned mouse mincle, the human mincle can act as a negative regulatory factor to inhibit immune responses. *Fonsecaea monophora* is a causative agent of chronic fungal skin infection. The β-glucan on *F. monophora* cell wall can trigger the host lectin Dectin-1 on the surface of dendritic cells (DCs). Then Dectin-1 activates the transcription factor interferon regulatory factor 1 (IRF1), which is vital for *IL-12A* transcription, and consequently triggers T_H_1 and T_H_17 immune responses against the fungus [40,223,224]. However, in this process, the human mincle on the surface of DCs is simultaneously triggered by the pathogen via proper carbohydrate molecule(s). Activated mincle induces degradation of IRF1 in the nucleus through an E3 ubiquitin ligase Mdm2-dependent degradation pathway. This process is deleterious for antifungal defense, as it leads to the loss of nuclear IRF1 activity and blocks *IL-12A* transcription [40].

Host lectins may act as spies to help pathogen transmission and dissemination. During the host antiviral response, the lectin DC-SIGN on the DCs surface binds the virus surface glycan, and subsequently “eats” and “digests” the virus by endocytosis and lysosomes [225]. Alternatively, after DCs catch the virus it migrates to lymphoid organs where DCs present the antigens of the virus to CD4^+^ T cells, and then trigger the adaptive immune response to effectively prevent virus infection [226,227,228]. This mechanism can be also utilized by some viruses. For instance, the host expressing DC-SIGN can enhance the transmission and dissemination of human immunodeficiency virus type 1 (HIV-1). HIV-1 causes acquired immune deficiency syndrome in humans by targeting CD4^+^ T cells [229,230]. In the early stages of infection, this targeting is less efficient, as the concentration for virus or CD4^+^ T cells is often very low at the infection sites [231]. Once HIV-1 enters the host, even in a very low concentration, it will be immediately captured by DCs via DC-SIGN [41]. This case is different from DC-SIGN mediated “eat” and “digest”, instead DC-SIGN can enrich the virus on the DCs surface and helps to retain HIV-1 infectivity [232]. Afterward, DCs carry the enriched HIV-1 to the lymphoid organs which rich in CD4^+^ T cells, and finally HIV-1 is enriched and transmitted to target cells [233,234,235]. In addition, DC-SIGN can also interact with Dengue virus [233], Hepatitis C virus [236], Sindbis virus [237], and the West Nile virus [42] via glycans that present on viral envelope glycoproteins and then participate in virus attachment to DCs for an efficiency infection.

## 8. Conclusions

**Figure 2:**
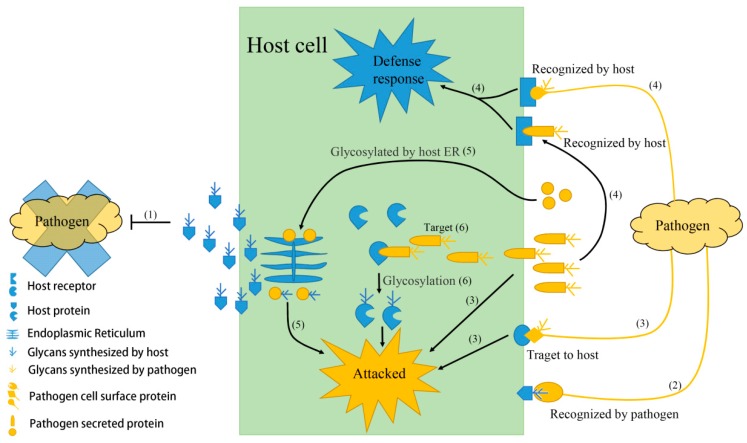
Schematic depicting the multiple functions of glycoproteins in host-pathogen interaction. (**1**) Glycoproteins surround the host cell act as barriers to inhibit pathogen adhesion, growth or kill pathogens; (**2**) alternatively these glycoproteins are recognized by pathogens to promote infection; (**3**) pathogen cell-surface or secreted glycoproteins directly cause disease; (**4**) alternatively these pathogen glycoproteins are recognized by host receptors to trigger defense responses; (**5**) pathogens also secrete non-glycoproteins to hosts and, subsequently be glycosylated in host endoplasmic reticulum (ER) before they turn pathogenic; (**6**) proteins secreted from pathogens can target and glycosylate key proteins of host intracellular signal pathway to cause disease.

Protein is an indispensable constituent of organism and plays a wide spectrum of functions in an organism. Generally, proteins need undergo different types of post-translational modifications for maturation and activation, and glycosylation is one of the most important among them [10]. It is employed mainly in six aspects in the host-pathogen interaction: (I) host glycoproteins separate pathogens from host as barriers; (II) host glycoproteins entrap the pathogen by specific interaction via carbohydrates with adhesins of the pathogen; (III) pathogen glycoproteins act as weapons to attack the host; (IV) hosts sense pathogen glycoproteins to induce resistance; (V) pathogen proteins are glycosylated in the host ER and turn pathogenic; (VI) pathogen proteins can glycosylate host proteins to cause disease (Figure 2). The activities of glycosylation on the host-pathogen interaction are attributed to the glycans that frequently alter the properties of proteins. The spatial structure of carbohydrate residues affects the conformation of proteins and masks specific sites of proteins or the hydrophilia of carbohydrate residues. The protein solubility is subsequently affected, resulting in a change of the protein activity, stability, or antigenicity. The glycans also act as nutrients for microbes or a mimic of cell wall components to directly inhibit pathogens. Investigating the role of protein glycosylation in host-pathogen interaction is significant in basic science since it provides a better understanding of host-pathogen interaction mechanism and its evolutionary inference, while it also sounds in applied science as it can be used to vaccine development, disease diagnosis, and drug therapy.

## Figures and Tables

**Table 1:** Glycoproteins discussed in this review.

Glycoprotein	Organism	Role of Protein Glycosylation	Glycoprotein Subtypes	Reference
Mucins (Muc1, Muc2, Muc5AC Muc6 etc.)	*Homo sapiens, Mus musculus*	Glycan-mediated adhesion, colonization and immune response of pathogens	*O*-linkage	[13,14,15,16,17]
Hydroxyproline-rich glycoproteins (HRGPs)	*Lycopersicon sculentum*, *Arabidopsis thaliana*, *Boswellia serrata* and *Boswellia carteri*	Affect HRGPs intra- and inter-molecular cross-linking		[18]
StAPs	*Solanum tuberosum*	Stabilization and activation of proteins	*N*-linkage	[19]
HMW1	*Haemophilus influenzae*			[20]
Slp1	*Magnaporthe oryzae*			[21]
BSA4				[22]
CBH1	*Trichoderma reesei*			[23]
PCIPG2	*Phytophthora capsici*			[24]
MgGPP	*Meloidogyne graminicola*			[25]
GrCLE	*Globodera rostochiensis*			[26]
Rho GTPase	*Homo sapiens*	Inactivation of proteins		[27]
Hc-CPL-1	*Haemonchus contortus*	Impact on protein stability and antigenicity		[28]
gp120	Human Immunodeficiency virus			[29]
E1	Semliki Forest virus			[30]
Hemagglutinin	Influenza virus			[31]
LecA	*Pseudomonas aeruginosa*	Stabilization and activation of proteins		[32]
LecB				
Bab A	*Helicobacter pylori*			[33]
Lab A				[17]
ECP6	*Cladosporium fulvum*			[34]
LysM1	*Trichophyton rubrum*			[35]
PSL	*Pisum sativum*			[36]
BpLec	*Bothrops pauloensis*			[37]
Mincle	*Homo sapiens*, *Mus musculus*			[38,39]
Dectin-1	*Homo sapiens*			[40]
DC-SIGN				[41,42]

**Table 2:** The lectin families in animals and plants.

Family	Subcellular Localization	Carbohydrate-Binding Specificity	Main Function	Reference
Animals				
Calnexins	Endoplasmic reticulum (ER), cell membrane	Glc_1_Man_9_ oligosaccharide	Molecular chaperones during glycoprotein synthesis	[121,122,123]
L-type lectins	ER, Golgi, ER-Golgi intermediate compartment	High-mannose *N*-glycans	Protein sorting in the endoplasmic reticulum	[124,125]
P-type lectins	Cell membrane, trans-Golgi network, endosomes	6-phosphorylated mannose	Intracellular routing of glycoconjugates	[126,127,128,129,130]
C-type lectins	Cell membrane, extracellular	*N*- or *O*-glycans	Cell adhesion, glycoprotein clearance, and innate immunity	[39,131]
Galectins (S-type lectins)	Cytoplasm, cell membrane, nuclear extracellular	Galactose, GalNAc, mannose	Cellular growth regulation and extracellular molecular bridging	[72,132,133,134,135,136]
I-type lectins	Cell membrane	Sialic acid, High mannose *N*-linked on L1 (cis) *N*-linked phosphacan, *N*-glycans contain fucose	Cell adhesion	[137,138,139]
R-type lectins	Golgi, cell membrane	Galactose, GalNAc	Enzyme targeting, glycoprotein hormone turnover.	[140,141,142]
F-box lectins	Cytoplasm	GlcNAc_2_	Degradation of misfolded glycoproteins.	[143]
Ficolins	Cell membrane, extracellular	GlcNAc, GalNAc	Self/non-self recognition	[144,145]
Chitinase-like lectins	Cell membrane, extracellular	Chito-oligosaccharides	Development, tissue remodelling and inflammation	[146,147,148]
F-type lectins (fucolectins)	Extracellular	Glycans terminal with fucose	Innate immunity	[149,150]
Intelectins	Extracellular, cell membrane	Gal, galactofuranose, pentoses	Fertilization and embryogenesis.	[151,152,153]
Plants				
*Agaricus bisporus* agglutinin homologs	Nucleus, cytoplasm, cell wall	Glycans contain Gal or/and GalNAc	Undetermined	[154,155,156]
Amaranthin	Nucleus, cytoplasm	Gal-β(1,3) GalNAc	Anti phytophagous and/or herbivorous animals	[157,158,159]
Chitinase-like lectins	Undetermined	High mannose *N*-glycans comprising the proximal pentasaccharide core structure	Defense response and host-microbe interaction	[160,161]
Cyanovirin-N	Undetermined	High-mannose type *N*-glycans	Undetermined	[162]
Euonymus lectin	Nucleus, cytoplasm	Blood group B oligosaccharides, high-mannose *N*-glycans	Regulate gene expression	[163,164,165]
*Galanthus nivalis* agglutinin	Nucleus, cytoplasm vacuolar	Mannose, oligomannosides, high-mannose and/or complex type *N*-glycans	Undetermined	[166,167,168]
Hevein	Vacuolar, cell wall	Chito-oligosaccharides, high mannose and/or complex *N*-glycans, *N*-acetyl-d-glucosamine	Anti phytophagous and/or herbivorous animals	[161,169,170,171]
Jacalins	Nucleus, cytoplasm, vacuole	Mannose, galactose	Development and defense response	[172,173,174,175]
Legume lectin	Extracellular, cytoplasm, vacuolar	Sialic acid, mannose, *N*-acetylgalactosamine,	Anti phytophagous and/or herbivorous animals, defense response	[176,177,178,179]
Lysin domain	Cell membrane, vacuolar	*N*-acetyl-d-glucosamine	Perception and recognition of pathogens	[21,180,181,182,183]
Nictaba	Nucleus, cytoplasm,	*N*- and *O*-glycans contain *N*-acetyllactosamine, high-mannose *N*-glycans	Anti phytophagous and/or herbivorous animals and regulate gene expression	[184,185,186]
Ricin-B family	Nucleus, cytoplasm, vacuolar	Gal, GalNAc, glycans contain sialic acid	Anti phytophagous and/or herbivorous animals	[187,188]
